# Dutch women in midwife-led care at the onset of labour: which pain relief do they prefer and what do they use?

**DOI:** 10.1186/1471-2393-13-230

**Published:** 2013-12-10

**Authors:** Trudy Klomp, Ank de Jonge, Eileen K Hutton, Antoine LM Lagro-Janssen

**Affiliations:** 1 Department of Midwifery Science. AVAG and EMGO Institute for Health and Care Research, VU University Medical Centre Amsterdam, D4445, Van der Boechorststraat 7, Amsterdam, NL 1081BT, Netherlands; 2 Midwifery Education Program, McMaster University Hamilton, Hamilton, Ontario, Canada; 3 Department of Primary Care and Community Care, Women’s Studies Medicine Radboud University Medical Centre Nijmegen, Nijmegen, Netherlands

## Abstract

**Background:**

Pain experienced during labour is more extreme than many other types of physical pain. Many pregnant women are concerned about labour pain and about how they can deal with this pain effectively.

The aim of this study was to examine the associations among low risk pregnant women’s characteristics and their preferred use and actual use of pain medication during labour.

**Methods:**

Our study is part of the DELIVER study: a dynamic prospective multi-centre cohort study. The data for this study were collected between September 2009 and March 2011, from women at 20 midwifery practices throughout the Netherlands. Inclusion criteria for women were: singleton pregnancies, in midwife–led care at the onset of labour and speaking Dutch, English, Turkish or Arabic. Our study sample consisted of 1511 women in primary care who completed both questionnaire two (from 34 weeks of pregnancy up to birth) and questionnaire three (around six week post partum). These questionnaires were presented either online or on paper.

**Results:**

Fifteen hundred and eleven women participated. Prenatally, 15.9% of women preferred some method of medicinal pain relief. During labour 15.2% of the total sample used medicinal pain relief and 25.3% of the women who indicated a preference to use medicinal pain relief during pregnancy, used pain medication. Non-Dutch ethnic background and planned hospital birth were associated with indicating a preference for medicinal pain relief during pregnancy. Primiparous and planned hospital birth were associated with actual use of the preferred method of medicinal pain relief during labour. Furthermore, we found that 85.5% of women who indicated a preference not to use pain medication prenatally, did not use any medication.

**Conclusions:**

Only a small minority of women had a preference for intrapartum pain medication prenatally. Most women did not receive medicinal pain relief during labour, even if they had indicated a preference for it.

Care providers should discuss the unpredictability of the labour process and the fact that actual use of pain medication often does not match with women’s preference prenatally.

## Background

Pain experienced during labour is a complex, subjective and multidimensional phenomenon. Aside from sensory components, it involves major emotional, motivational and cognitive dimensions [1,2]. Labour pain is more extreme than many other types of physical pain [3,4] and many pregnant women are concerned about the pain of labour and about how they can deal with it effectively [4]. On the other hand, women have also described their experience of giving birth as an empowering experience which gave them a sense of pride in their ability to deal with the pain [5,6]. Labour pain can be managed through medicinal and non-medicinal approaches. Non-medicinal methods of pain relief include relaxation techniques, distraction techniques and continuous support [7-9]. Epidural analgesia, pethidine or morphine injections, and remifentanil infusions are examples of medicinal pain relief [9]. Christiansen et al. [10] and Hodnett et al. [11] reported an association between involvement in decision making and satisfaction with the experience of childbirth. Involvement in decision making and the ability to choose between different methods of pain relief contributes to childbirth satisfaction [12].

In recent years there has been an increase in the number of women opting for epidural analgesia during labour [13,14]. The use of some method of medicinal pain relief has become standard procedure in many developed countries [15,16]. The Netherlands has a community-based maternity care system, with approximately 84% of all pregnancies starting in midwife-led care [17]. Low-risk women in midwife-led care may choose to give birth at home, in a birth centre or in hospital with their own midwife. If risk factors or complications arise, women are referred to obstetrician-led care. Medical interventions such as medicinal pain relief, electronic foetal monitoring and augmentation of labour only take place in obstetrician-led care. Women who fear labour pain and who have decided that they will choose for medicinal pain relief before going into labour may be referred by their midwife for a consultation with the obstetrician in order to discuss about their labour pain management. However, usually these women will start their labour in midwife-led care and they will make arrangements with their midwives that they will be referred for pain medication as soon as labour starts [18].

The Dutch guideline concerning medicinal pain relief was introduced in 2008 [19]. This guideline states that a woman’s request is a sufficient medical indication for medicinal pain relief during labour, and that epidural analgesia should be the method of choice for the elimination of labour pain. Despite the Dutch tradition of a ‘natural’ birth without medicinal pain relief, the number of women using medicinal pain relief in this context is increasing every year [17]; 13.9% of women without a primary caesarean section used epidural analgesia in 2009 [17].

Little is known about pregnant women’s prenatal preference regarding pain relief and their actual pain relief in the Netherlands during labour. In addition, little is known about women’s socio-demographic and personal characteristics that are associated with a preference for medicinal pain relief during pregnancy.

The aim of this study was to examine the associations between women’s characteristics and their preferred use and actual use of pain medication during labour.

## Methods

### Study population

Our study was part of the DELIVER study: a dynamic prospective multi-centre cohort study [20]. This study was approved by the Medical Ethical Committee of VU University Medical Center Amsterdam (VUmc). The data for this study were collected between September 2009 and March 2011, from women at 20 midwifery practices throughout the Netherlands.

We approached twenty of the 519 primary care practices in the Netherlands and invited them to participate in this study. We purposively selected practices using three stratification criteria: region: north, middle, south; level of urbanisation: urban, rural or combined urban/rural; practice type: dual or group practice (Table 1). The approached practices received a brochure with information on the study and were visited by two members of the DELIVER research team who explained the study in further detail. If a practice declined participation, a replacement was found taking region, urbanisation and practice type into account. Ultimately, fourteen practices declined participation, mostly because of time constraints. Midwives invited all women in their practices who spoke Dutch, English, Turkish or Arabic. Those pregnant women who were prepared to participate in the study gave informed consent to their midwife. For the purposes of the study, these women received three questionnaires: the first early in pregnancy (at around 12 weeks), the second between 34 weeks of pregnancy and birth. and the third at around six weeks post partum. Depending on the preferences of the women, these questionnaires were presented either online or on paper. In an attempt to boost the response rate, successive reminders were sent to non-responders one week after the initial invitation, and student-assistants called non-responders between three to four weeks of non-responding. Non-responders from other cultural backgrounds were offered an opportunity to participate in the study by means of a telephone interview in Dutch, Turkish, Berber or Arabic (depending on their preference). The DELIVER client data were linked to primary care data from the Netherlands Perinatal Register (‘Landelijke Verloskundige Registratie’. LVR1).

**Table 1:** Characteristics of the 20 midwifery practices

**Practice**	**Region**	**Level of urbanisation**	**Practise type (n = number of practising midwives)**
1	South	Rural/Urban	Group (4)
2	South	Rural/Urban	Group (6)
3	Centre	Rural/Urban	Group (7)
4	North	Rural	Group (3)
5	Centre	Urban	Group (5)
6	Centre	Rural	Group (5)
7	North	Urban	Group (3)
8	North	Rural	Group (4)
9	South	Rural/Urban	Group (5)
10	Centre	Rural/Urban	Group (6)
11	North	Rural	Duo (2)
12	North	Urban	Group (4)
13	Centre	Rural/Urban	Group (5)
14	Centre	Rural	Group (6)
15	Centre	Rural/Urban	Group (5)
16	North	Rural	Group (3)
17	Centre	Rural/Urban	Group (5)
18	Centre	Urban	Group (5)
19	South	Rural/Urban	Duo (2)
20	Centre	Urban	Group (6)

For this study, all women with singleton pregnancies who were in midwife–led care at the onset of labour and who completed both questionnaires two (from 34 weeks of pregnancy until delivery) and three (around six weeks after delivery) were selected. We excluded women who did not meet the criteria for midwife-led care at the onset of labour. Thus we excluded women who were referred to obstetrician-led care during pregnancy; gave birth before 37 weeks and 0 days or after 42 weeks and 0 days gestation and were referred for prolonged rupture of membranes (> 24 hrs without being in active labour). Women who had an induction of labour or planned Caesarean section start labour in obstetrician-led care and were therefore not included in our sample.

### The variables used in the study

Data of socio-demographic and personal characteristics were used in the analyses as independent variables. Based on prior studies, we used five variables known to be associated with medicinal pain management use; age, level of education, ethnic background, parity and planned place of birth [21-23].

Women reported their date of birth; age was subsequently categorized into ‘under 25’, ‘from 25 to 35’ and ‘over 35’. Women’s highest level of education was recoded into low (no education, only primary education or lower vocational education), medium (only secondary school education or medium vocational education) and high (college, university or post-graduate education). Women were asked about the country of birth of both parents. Women’s ethnicity was based on the definition used by Statistics Netherlands [24], which considers someone to be of non-Dutch ethnicity if at least one of the parents was born in a country other than the Netherlands. If the parents were born in two different countries, then the mother’s country of birth is considered the ‘country of origin’. Finally, women reported their number of children, which was then dichotomized into ‘primiparous’ and ‘parous’.

Planned place of birth (home or hospital) was taken from the perinatal registration form of the Netherlands Perinatal Registry which was filled in by the midwife during pregnancy*.*

In the prenatal questionnaire, women were asked whether they had a preference in terms of pain management during labour and, if so, what would be their preference in terms of medication; pethidine, remifentanyl, epidural or no medication (Additional file 1). In the questionnaire, women were informed that they would have to be referred to obstetrician-led care if they would choose to use medicinal pain relief. In the postnatal questionnaire, women were asked whether they used any method of medicinal pain relief during labour and, if so, what method of medication: pethidine, remifentanyl, epidural or no medication (Additional file 2).

For the analyses regarding women who used their preferred method of medicinal pain relief, age and education were dichotomised because of limited numbers in some categories (age: ≤35, >35 and education: low/medium, high).

Women who had a preference for medicinal pain relief were compared with women who did not have a preference for medicinal pain relief. The following three groups were created for the analysis regarding women who used their preferred method of pain relief: no medication; epidural and pethidine or remifentanil. Women who used epidural in combination with pethidine or remifentanil were placed in the epidural group. For the multivariable analyses, women who used any form of pain medication were combined as one group.

### Statistical analyses

We used descriptive statistical methods to determine frequencies and percentages. Univariable logistic regression methods were used to calculate crude odds ratios and multivariable logistic regression methods for adjusted odds ratios with 95% confidence intervals. Because women in our study population were clustered into twenty different midwifery practices. We used multi-level analysis to control for the dependency of measurements within these practices. Except for multi-level analyses, all analyses were carried out in IBM SPSS, version 20. Multi-level analyses were carried out in Stata IC 20.

## Results

**Figure 1:**
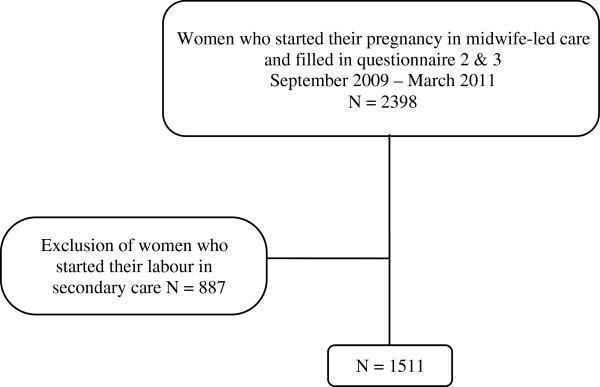
Flow diagram of women in midwife-led care.

The overall net response rate of the DELIVER study was 62% [20]. Of all 7685 women that participated in the DELIVER study, 3334 women completed the second questionnaire and 3952 completed the third questionnaire. The DELIVER client data were successfully linked in 86.3% of the cases with data from the Netherlands Perinatal Registry. Of all women who started their pregnancy in midwife-led care, 2398 individuals filled in both the second and third questionnaires. Of these, 1511 women started labour in midwife-led care (Figure 1). The characteristics of the women in the study are shown in Table 2. Highly educated women and those of Dutch ethnic background were over-represented in our study population compared to the overall Dutch perinatal registration of midwife-led care and obstetrician-led care in total (56.5% versus 48.2% and 88.5% versus 74.2% respectively).

**Table 2:** Study sample

**Characteristics of the study sample (N** = **1511)**			
	**N**	**%**	**PRN**^**a **^**data %**
***Age group (years)***			
<25	100	6.6	-
25–35	1191	78.8	-
>35	220	14.6	-
***Education level***			
Low	157	10.4	15.5
Medium	501	33.2	36.4
High	853	56.5	48.2
***Ethnic background***^***b***^	n = 1509		
Dutch	1336	88.5	74.2
Non - Dutch	173	11.5	20.8
***Planned place of birth***^***c***^	n = 1470		
Home	910	61.9	-
Hospital	565	38.1	-
***Parity***			
Nulliparous	686	45.4	45.8
Parous	825	54.6	54.2

^a^Data of the Dutch pregnant population (PRN. 2009).

^b^Missing ethnic background n = 2.

^c^Missing planned place of birth n = 41.

### Women’s preferences regarding medicinal pain relief

Prenatally, 15.9% of women preferred to use some method of medicinal pain relief (Table 3). Women with a non-Dutch background were more likely to prefer using medicinal pain relief than women with a Dutch background (OR 1.96 CI 1.31 to 2.94), and women with a planned hospital birth were more likely to prefer using a medicinal method of pain relief than women with a planned home birth (OR 3.37 CI 2.46 to 4.63) (Table 4).

**Table 3:** Women’s preferences* and women’s used pain relief

			**Used method of medicinal pain relief**		
			**Epidural**	**Pethidine or remifentanil**	**No medication**
		**No (%)**	**No (%)**	**No (%)**	**No (%)**
*Preference*	Medication	233 (15.9)	35 (15.0)	24 (10.3)	174 (74.7)
	No medication	1231 (84.1)	109 (8.9)	70 (5.7)	1052 (85.5)
	Total	1464	144 (9.8)	94 (6.4)	1226 (83.8)

*Missing ‘women’s preferences’ n = 47.

**Table 4:** **Association between age, education level, ethnicity, planned place of birth, parity and women’s preference to use medicinal pain (N** = **1511)**

	**Total N**^**b**^	**No (%)**	**Univariable OR (CI)**	**Multivariable**^**a**^**OR (CI)**
**Age groups (years)**				
*<25*	100	9 (9.2)	0.55 (0.27–1.11)	0.60 (0.29–1.27)
*25–35*	1191	181 (15.6)	1.0	1.0
*>35*	220	43 (20.2)	1.37 (0.95–1.99)	1.11 (0.74–1.67)
**Level of education**				
*Low*	157	22 (14.3)	0.97 (0.58–1.62)	0.93 (0.54–1.60)
*Medium*	501	72 (14.7)	1.0	1.0
*High*	853	139 (16.7)	1.17 (0.86–1.59)	1.11 (0.79–1.56)
**Ethnic background**^**c**^				
*Dutch*	1336	186 (14.2)	1.0	1.0
*Non-Dutch*	173	47 (28.8)	2.45 (1.69–3.56)**	1.96 (1.31–2.94)**
**Planned place of birth**^**d**^				
*Home*	910	85 (9.5)	1.0	1.0
*Hospital*	560	142 (26.2)	3.37 (2.51–4.52)**	3.37 (2.46–4.63)**
***Parity***				
*Primiparous*	686	108 (16.0)	1.03 (0.78–1.36)	0.90 (0.66–1.22)
*Parous*	825	125 (15.6)	1.0	1.0

^a^Adjusted for age, education, ethnic background, planned place of birth and parity.

^b^Missing ‘women’s preference to use medicinal pain relief n = 47.

^c^Missing ethnic background n = 2.

^d^Missing place of birth n = 41, **p < 0.05, R^2^ = 10%.

### Use of medicinal pain relief

Of the women who started labour in midwife-led care 16.2% of the women used some method of medicinal pain relief during labour, 9.8% used epidural analgesia; 6.4% used pethidine or remifentanil (Table 3). Of the women preferring no medication for pain relief prenatally, 85.5% used no medication. Of the women preferring medicinal pain relief 25.3% used medicinal pain relief (Table 3).

Women with a planned hospital birth who indicated a preference to use medicinal pain relief were more likely to use it than women with a planned home birth with the same preference (OR 2.14 CI 1.04 to 4.39). Primiparous women who indicated a preference to use medicinal pain relief were more likely to use it than parous women with the same preference (OR 4.60 CI 2.27 to 9.13) (Table 5).

**Table 5:** **Association between age, education level, ethnicity, planned place of birth parity, and use of medicinal pain relief method that was preferred prenatally (N** = **1511)**

	**Total N**^**a**^	**No (%)**	**Univariable OR (CI)**	**Multivariable**^**b **^**OR (CI)**
**Age groups (years)**				
*≤35*	1291	14 (32.6)	1.56 (0.76–3.20)	1.96 (0.87–4.43)
*>35*	220	45 (23.7)	1.0	1.0
**Level of education**				
*Low-Medium*	658	17 (18.1)	1.0	1.0
*High*	853	42 (30.2)	1.96 (1.04–3.71)**	1.66 (0.83–3.34)
**Ethnic background**^**c**^				
*Dutch*	1336	47 (25.3)	1.0	1.0
*Non-Dutch*	173	12 (25.5)	1.01 (0.49–2.11)	0.74 (0.33–1.68)
**Planned place of birth**^**d**^				
*Home*	910	17 (20.0)	1.0	1.0
*Hospital*	560	42 (29.6%)	1.68 (0.88–3.19)	2.14 (1.04–4.39)**
***Parity***				
*Primiparous*	686	41 (38.0)	3.64 (1.93–6.85)**	4.60 (2.27–9.13)**
*Parous*	825	18 (14.4)	1.0	1.0

^a^Missing ‘use of medicinal pain relief which was preferred prenatally’ n = 10, ^*b*^adjusted for age, education level, ethnic background, planned place of birth and parity, ^c^missing ‘ethnic background’ n = 2, ^d^missing ‘planned place of birth’ n = 41. **p < 0.05, R^2^ = 18%.

## Discussion

One of the main findings was that 85.5% of the women in our study indicated prenatally a preference to use no medication for pain relief during labour. Secondly, our study showed that women with a non-Dutch ethnic background were more likely to indicate a preference for medicinal pain relief prenatally compared to women with a Dutch ethnic background. Thirdly, our study found that women with a planned hospital birth were more likely to indicate a preference for medicinal pain relief compared to women with a planned home birth. Finally, our study showed that women with a planned hospital birth who preferred to use medicinal pain relief were more likely to use medicinal pain relief compared to women with a planned home birth. Primiparous women were more likely to use their preferred method of medicinal pain relief compared to parous women.

### Women’s preferences regarding medicinal pain relief

Despite the growing numbers of medicinal pain relief in labour worldwide and the introduction of guidelines that should ensure access to epidural analgesia for all Dutch women, most women in midwife-led care in our study still preferred prenatally not to use medicinal pain relief. This finding has not previously been reported. It might be that most women in midwife-led care with low-risk profiles believe they will have a natural birth which they can manage without medicinal pain relief. Another reason might be that the guideline of medicinal pain relief in labour, which was introduced in 2008, is not implemented in every midwifery practice [25]. This would mean that not all women are informed about their options regarding medicinal pain relief.

We found that women with a non-Dutch ethnic background were more likely to indicate a preference for, and to use the preferred medicinal pain relief. These women might be more accustomed to use medicinal pain relief in labour compared to women with a Dutch ethnic background because of the maternity culture in their country of origin [5,26,27]. It is also possible that women from non-Dutch cultures might have a more negative attitude towards labour pain [27].

We found that women with a planned hospital birth were more likely to indicate a preference to use medicinal pain relief compared to women with a planned home birth. Women who choose a planned hospital birth might feel less secure and more anxious around their ability to give birth ‘naturally’ without medicinal pain relief. Therefore it is more likely that these women would choose a hospital setting for birth so as to avoid transport from home to hospital in case they would need medicinal pain relief.

Surprisingly, 9.5% of the women with a planned home birth indicated a preference to use medicinal pain relief, even though this is never administered at home. It might be that women take into account different scenarios that may occur during labour. They might plan to stay at home without medicinal pain relief as long as labour progresses well. However, at the same time women might choose for medicinal pain relief if labour is more difficult than anticipated. This finding is in line with the interview study of Klomp et al. [18]. In this qualitative study most women indicated prenatally that they did not want to make use of medicinal pain relief during labour but at the same time they had thought of their preferred method in case they would need some pain medication after all.

### Use of medicinal pain relief

Other studies have suggested that the use of medicinal pain relief is not solely dependent on the preferences and backgrounds of the women in question; it also seems to depend on the culture of the maternity care system in the country, in the region or even at the individual delivery unit [26,27]. Christeans et al. [27] suggest that Dutch women have more positive attitudes towards labour pain compared to women in Belgium who have more negative attitudes. Our finding of relatively low actual use of some method of medicinal pain relief is consistent with these findings.

Surprisingly, only 25.3% of the women who indicated prenatally a preference to use medicinal pain relief during labour actually used a medicinal method. It might be that women’s preferences regarding medicinal pain relief are unmet by their care-providers. Although a multidisciplinary Dutch guideline states that women who request pain medication should receive this, it is possible that not all professionals adhere to this recommendation. Since research has shown that women’s involvement in decision making on the use of pain relief contributes to childbirth satisfaction [11], further studies are needed into the decision making process regarding pain relief in the Netherlands. On the other hand, it is also likely that women take into account different scenarios that may occur during labour as formulated before. Medicinal pain relief during labour does not seem to be a dichotomous choice for women but to comprise a continuum of choices. Furthermore, we found that 85.5% of women who indicated a preference to use no medication for pain relief prenatally, did not use it. These findings are in line with studies of Walsh & Devane [28] and Begley et al. [29] which found that women in midwife-led care during labour and birth use less medicinal pain relief compared to women in other models of care. All our women started their labour in midwife-led care.

Our study also showed that primiparous women who indicated a preference to use medicinal pain relief were more likely to use it than parous women. It might be that parous women are more likely to have a fast labour and therefore these women have little time and also feel less need to use their preferred medicinal pain relief.

Women with a planned hospital birth who indicated a preference to use medicinal pain relief were more likely to use it than women with a planned home birth. If women give birth in hospital medicinal pain relief is more readily available and it might be that these women are more likely to use their preferred method because of this availability [30,31].

### Limitations

The women in this study filled in the post partum questionnaire at different points in time from two weeks post partum until three months post partum. This study, therefore, does not take into account that some women may have changed their memories of the used method of pain relief in labour due to recall bias.

Due to the limited numbers of women in each different ethnic group we decided to dichotomize ethnic background into two groups: Dutch and non-Dutch. Further study is needed into the preferences and use of pain relief among different ethnic minority groups.

### Strengths

A major strength of our study is that women were asked to indicate their preferred method of pain relief before they went into labour and their used method of pain relief after they gave birth. In some studies [23,32] women were asked after birth which method of pain relief they preferred when they were still pregnant but experience of labour may have influenced women’s recall in these cases.

Our large study provides a good cross-sectional insight into the characteristics associated with women who indicate a preference for medicinal pain relief at some point between 35 weeks of pregnancy and start of labour and the characteristics of women who prefer to use and who used medicinal pain relief.

## Conclusions

Even though the prevalence of women preferring medicinal pain relief was low (15.9%), surprisingly, only one quarter of this group actually received pain medication. Of the women who did not indicate any preference for medicinal pain relief prenatally (84.1%) a small proportion (14.6%) used medicinal pain relief.

With regard to counselling for labour pain management, care providers should discuss the unpredictability of the labour process. Labour can be easier or more difficult than anticipated. This can help women to have realistic expectations towards labour pain management.

## Competing interests

The authors declare that they have no competing interests.

## Authors’ contributions

TK and AdJ designed the study of labour pain questionnaires as part of the DELIVER study. TK recruited the midwifery practices, Judith Manniën monitored data collection and TK supervised data collection. TK conducted data analyses and was also primarily responsible for data interpretation. TL, EH and AdJ assisted with data interpretation. All authors read and corrected draft version of the manuscript and approved the final manuscript.

## Pre-publication history

The pre-publication history for this paper can be accessed here:

http://www.biomedcentral.com/1471-2393/13/230/prepub

## Supplementary Material

Additional file 1 DELIVER women questionnaire 2 (>34 weeks – < date of birth). Click here for file

Additional file 2 DELIVER women questionnaire 3 (around six weeks post partum). Click here for file

## References

[B1] Melzack R From the gate to the neuromatrix Pain 1999 13 S121 S126 10491980 10.1016/S0304-3959(99)00145-1

[B2] Lowe NK The nature of labor pain Am J Obstet Gynecol 2002 13 S16 S24 12011870 10.1067/mob.2002.121427

[B3] Niven C Psychological Care for Families: Before, During and After Birth 1992 Oxford: Butterworth Heinemann

[B4] Lally JE Murtagh MJ Macphail S Thomson R More in hope than expectation: a systematic review of women’s expectations and experience of pain relief in labour BMC Med 2008 13 7 10.1186/1741-7015-6-7 18366632 PMC2358911

[B5] de Vries R A Pleasing Birth: Midwifery and Maternity Care in the Netherlands 2005 Amsterdam: University Press

[B6] Hayes-Klein H Hayes-Klein H Introduction: my story Conference papers/Human rights in childbirth, International conference of jurists, midwives & obstetricians 2012 The Netherlands: Bynkers Hoek, The Hageue 11 21

[B7] Anim-Somuah M Smyth RM Jones L Epidural versus non-epidural or no analgesia in labour Cochrane Database Syst Rev 2011 13 CD000331 10.1002/14651858.CD000331.pub3 22161362

[B8] Hutton EK Kasperink M Rutten M Reitsma A Wainman B Sterile water injection for labour pain: a systematic review and meta-analysis of randomised controlled trials BJOG 2009 13 1158 1166 10.1111/j.1471-0528.2009.02221.x 19459860

[B9] Jones L Othman M Dowswell T Alfirevic Z Gates S Newburn M Pain management for women in labour: an overview of systematic reviews Cochrane Database Syst Rev 2012 13 CD009234 10.1002/14651858.CD009234.pub2 PMC7132546 22419342

[B10] Christiansen P Klostergaard KM Terp MR Poulsen C Agger AO Rasmussen KL [Long-memory of labor pain] Ugeskr Laeger 2002 13 4927 4929 12416074

[B11] Hodnett ED Pain and women’s satisfaction with the experience of childbirth: a systematic review Am J Obstet Gynecol 2002 13 S160 S172 12011880 10.1067/mob.2002.121141

[B12] Hodnett ED Gates S Hofmeyr GJ Sakala C Weston J Continuous support for women during childbirth Cochrane Database Syst Rev 2011 13 CD003766 10.1002/14651858.CD003766.pub3 21328263

[B13] Christiaens W Nieuwenhuijze MJ de Vries R Trends in the medicalisation of childbirth in Flanders and the Netherlands Midwifery 2013 13 e1 e8 10.1016/j.midw.2012.08.010 23266221

[B14] Osterman MJ Martin JA Epidural and spinal anesthesia use during labor: 27-state reporting area, 2008 Natl Vital Stat Rep 2011 13 1 13 16 21553556

[B15] Marmor TR Krol DM Labor pain management in the United States: understanding patterns and the issue of choice Am J Obstet Gynecol 2002 13 S173 S180 12011881 10.1067/mob.2002.121258

[B16] Van den Bussche E Crombez G Eccleston C Sullivan MJ Why women prefer epidural analgesia during childbirth: the role of beliefs about epidural analgesia and pain catastrophizing Eur J Pain 2007 13 275 282 10.1016/j.ejpain.2006.03.002 16624602

[B17] PRN The Netherlands perinatal registry Perinatale zorg in Nederland 2001–2009 http://www.perinatreg.nl/jaarboeken_zorg_in_nederland

[B18] Klomp T Mannien J De JA Hutton EK Lagro-Janssen AL What do midwives need to know about approaches of women towards labour pain management? A qualitative interview study into expectations of management of labour pain for pregnant women receiving midwife-led care in the Netherlands Midwifery 2013 doi:pii: S0266-6138(13)00153-8. 10.1016/j.midw.2013.04.013. [Epub ahead of print] 10.1016/j.midw.2013.04.013 23790961

[B19] CBO Guideline pharmacological pain relief during labour (Richtlijn Medicamenteuze Pijnbehandeling tijdens de bevalling) 2008 Utrecht: Dutch Organisation of Anaesthesiology (NVA) and Dutch Organisation of Obstetrics and Gynaecology (NVOG)

[B20] Mannien J Klomp T Wiegers T Pereboom M de JA Evaluation of primary care midwifery in The Netherlands: design and rationale of a dynamic cohort study (DELIVER) BMC Health Serv Res 2012 13 69 10.1186/1472-6963-12-69 22433820 PMC3331850

[B21] Le Ray C Goffinet F Palot M Garel M Blondel B Factors associated with the choice of delivery without epidural analgesia in women at low risk in France Birth 2008 13 171 178 10.1111/j.1523-536X.2008.00237.x 18844642

[B22] Jimenez-Puente A Benitez-Parejo N Del Diego-Salas J Rivas-Ruiz F Maanon-Di LC Ethnic differences in the use of intrapartum epidural analgesia BMC Health Serv Res 2012 13 207 10.1186/1472-6963-12-207 22818255 PMC3411410

[B23] Green JM Expectations and experiences of pain in labor: findings from a large prospective study Birth 1993 13 65 72 10.1111/j.1523-536X.1993.tb00419.x 8240609

[B24] Statistics Netherlands (CBS) 2013 http://www.cbs.nl/en-GB/menu/themas/dossiers/allochtonen/nieuws/default.htm

[B25] Walraven C Review: the effect of audit and feedback on guideline adherence and patient outcomes is limited Ann Intern Med 2013 13 JC11 23420248 10.7326/0003-4819-158-4-201302190-02011

[B26] Schytt E Waldenstrom U Epidural analgesia for labor pain: whose choice? Acta Obstet Gynecol Scand 2010 13 238 242 10.3109/00016340903280974 19824867

[B27] Christiaens W Verhaeghe M Bracke P Pain acceptance and personal control in pain relief in two maternity care models: a cross-national comparison of Belgium and the Netherlands BMC Health Serv Res 2010 13 268 10.1186/1472-6963-10-268 20831798 PMC2944275

[B28] Walsh D Devane D A metasynthesis of midwife-led care Qual Health Res 2012 13 897 910 10.1177/1049732312440330 22427456

[B29] Begley C Devane D Clarke M McCann C Hughes P Reilly M Comparison of midwife-led and consultant-led care of healthy women at low risk of childbirth complications in the Republic of Ireland: a randomised trial BMC Pregnancy Childbirth 2011 13 85 10.1186/1471-2393-11-85 22035427 PMC3226589

[B30] Gottvall K Waldenstrom U Tingstig C Grunewald C In-hospital birth center with the same medical guidelines as standard care: a comparative study of obstetric interventions and outcomes Birth 2011 13 120 128 10.1111/j.1523-536X.2010.00461.x 21599734

[B31] Pavlova M Hendrix M Nouwens E Nijhuis J van MG The choice of obstetric care by low-risk pregnant women in the Netherlands: implications for policy and management Health Policy 2009 13 27 34 10.1016/j.healthpol.2009.05.008 19540012

[B32] Scotland GS McNamee P Cheyne H Hundley V Barnett C Women’s preferences for aspects of labor management: results from a discrete choice experiment Birth 2011 13 36 46 10.1111/j.1523-536X.2010.00447.x 21332773

