# Supplementary material for: Dutch women in midwife-led care at the onset of labour: which pain relief do they prefer and what do they use?
Source: BMC Pregnancy Childbirth. 2013 Dec 10;13:230. doi: 10.1186/1471-2393-13-230 (PMC4029565; doi:10.1186/1471-2393-13-230)
Supplement: Additional file 1 — DELIVER women questionnaire 2 (>34 weeks – < date of birth). [file 1471-2393-13-230-S1.doc]

**Additional file 1 - DELIVER** **women questionnaire 2 (>34 weeks – < date of birth)**

1. Do you have any preference regarding labour pain management?
 0 No → please continue to question 3 and further
 0 Yes

2. What would be your preference in terms of pain medication? (if you would use pain medication during labour you have to be referred to obstetrician led care in hospital)
 0 Injection with medicinal pain relief (pethidine or morphine)
 0 Self controlled drip with medicinal pain relief (remifentanyl)
 0 Low back drip with the option of self-control (epidural)
 0 No medication
