# Supplementary material for: Dutch women in midwife-led care at the onset of labour: which pain relief do they prefer and what do they use?
Source: BMC Pregnancy Childbirth. 2013 Dec 10;13:230. doi: 10.1186/1471-2393-13-230 (PMC4029565; doi:10.1186/1471-2393-13-230)
Supplement: Additional file 2 — DELIVER women questionnaire 3 (around six weeks post partum). [file 1471-2393-13-230-S2.doc]

**Additional file 2 - DELIVER** **women questionnaire 3 (around six weeks post partum)**

1. Did you use any method of medicinal pain relief during labour?
 0 No → please continue to question 2 and further
 0 Yes → What method of medicinal pain relief was used? (*You may select more than one answer*)
 0 Injection with medicinal pain relief (pethidine or morphine)
 0 Self controlled drip with medicinal pain relief (remifentanyl)
 0 Low back drip with the option of self-control (epidural)
 0 General anaesthetic
